# Comparative metabolomic analyses of *Dendrobium officinale Kimura et Migo* responding to UV-B radiation reveal variations in the metabolisms associated with its bioactive ingredients

**DOI:** 10.7717/peerj.9107

**Published:** 2020-06-29

**Authors:** Yue Chen, Qi Shen, Ping Lv, Chongbo Sun

**Affiliations:** 1 Institute of Horticulture, Zhejiang Academy of Agriculture Science, Hangzhou, Zhejiang, China; 2 Key Laboratory of Creative Agriculture, Ministry of Agriculture, Hangzhou, China; 3 Plant Protection and Microbiology, Zhejiang Academy of Agriculture Science, Hangzhou, Zhejiang, China; 4 Agro Technical Extension and Service Center, Hangzhou, China

**Keywords:** Active ingredient, Dendrobium, Metabolome, Metabolite, UV-B

## Abstract

**Background:**

*Dendrobium officinale Kimura et Migo*, a member of the genus *Dendrobium*, is a traditional Chinese medicine with high commercial value. The positive roles of UV-B radiation on active ingredient metabolism in various medicinal plants have been studied. However, the metabolic responses of *D. officinale* stems to UV-B treatment is largely unknown.

**Methods:**

An untargeted metabolomics method was used to investigate the metabolic variations in* D. officinale* stems between the control and UV-B treatments.

**Results:**

In total, 3,655 annotated metabolites, including 640 up- and 783 down-regulated metabolites, were identified and grouped into various primary metabolic categories. Then, a number of metabolites involved in the polysaccharide, alkaloid and flavonoid biosynthesis pathways were identified. For polysaccharide biosynthesis, several intermediate products, such as pyruvate, secologanate, tryptophan and secologanin, were significantly up-regulated by the UV-B treatment. For polysaccharide biosynthesis, many key fundamental building blocks, from the glycolysis, starch and sucrose metabolism, and fructose and mannose metabolism pathways, were induced by the UV-B treatment. For flavonoid metabolism, accumulations of several intermediate products of chalcone synthase, chalcone isomerase and flavanone 3-hydroxylase were affected by the UV-B treatment, indicating an involvement of UV-B in flavonoid biosynthesis. The UV-B induced accumulation of polysaccharides, alkaloids and flavonoids was confirmed by HPLC analysis. Our study will help to understand the effects of UV-B on the accumulation of active ingredients in *D. officinale*.

## Introduction

*Dendrobium officinale*, an important member of the *Dendrobium* genus, is greatly prized medicinal herb in East Asian countries (Meng et al., 2016). In China, the stems of *D. officinale*, also named as ‘Fengdou’, have been treated as herb medicine for antipyretic and immune regulatory for a thousand years (Yang, Wang & Xu, 2006). In the past decades, destructive collection has driven the wild *D. officinale* plants to the edge of extinction (Hui & Yang, 2006).

Many extracted ingredients from *D. officinale* stems are secondary metabolites, such as polysaccharides, alkaloids, terpenes and flavonoids (Shen et al., 2017; Yang et al., 2017; Zhang et al., 2016). Previous works reported that abundant soluble polysaccharides were enriched in the stems of *D. officinale*. Analysis data showed that most soluble polysaccharides in the stems of *D. officinale* were identified as glucomannan, which consist of glucose and mannose. For example, 2,3-O-acetylated-1,4-β-d-glucomannan (DOP-1-1) is a polysaccharide isolated from the stem of *D. officinale* (Huang et al., 2018). Glucomannan has been demonstrated to have prominent bioactivities, such as anti-oxidant, immune regulation, and antitumor (Wang et al., 2010a). For example, DOP-GY, a kind of polysaccharide from *D. officinale*, played important roles in the amelioration of H_2_O_2_-mediated apoptosis in H9c2 cardiomyocytes *via* the MAPK and PI3K/AKT pathways (Zhang et al., 2017). Further structural characterization showed that a neutral hetero-polysaccharide from *D. officinale*, DOP-1-1, consisted of glucose and mannose (1: 5.9) with a large molecular weight (He et al., 2016). To date, more and more novel polysaccharides from *D. officinale* have been identified, allowing the content of polysaccharides a major quality indicator of ‘Fengdou’ (Huang et al., 2016; Xie et al., 2016).

As another major class of active ingredients, alkaloids from *D. officinale* have been isolated and clarified. Although the content of total alkaloids in *D. officinale* is extremely low, their protective effects on memory impairment have been well-studied (Li et al., 2011). Additionally, alkaloids also exhibit a degree of antioxidant, and anticancer activities in both vitro and in vivo investigations (Wang et al., 2010b). The major constituent of alkaloids in *D. officinale* was classified into the terpenoid indole alkaloid (TIA) category (Chen, Xiao & Guo, 2006; Guo et al., 2013). According to the model plants, there is a conserved upstream pathway of TIA biosynthesis, which generates a strictosidine backbone as an intermediate of alkaloid biosynthesis (Li et al., 2015; Miralpeix et al., 2014). A potential TIA biosynthesis pathway in *D. officinale* involved in a number of metabolites from the mevalonate (MVA), methylerythritol phosphate (MEP), and monoterpenoid biosynthesis pathways (Guo et al., 2013; Shen et al., 2017). However, the biosynthesis pathway of alkaloids following strictosidine remain poorly understood in *D. officinale*.

Flavonoids are ubiquitous phytochemical ingredients with diverse biological functions and play essential roles in pharmaceutical industry (Cao et al., 2013; Hao et al., 2017). Anthocyanins greatly accumulates in the stems of *D. officinale*, providing a foundation for producing functional foods (Yu et al., 2018b). Simultaneous qualitative and quantitative analyses of flavonoid glycosides is another effective method to authenticate and evaluate *D. officinale* from different regions (Zhou et al., 2018). Differential fingerprints of flavonoid can be used to discriminate *D. officinale* from other *Dendrobium* plants (Ye et al., 2017).

Ultraviolet-B (UV-B) is a key component of solar radiation and plays a role in secondary metabolites accumulation in different species (Mewis et al., 2012; Takshak & Agrawal, 2016). In the industrial field, UV-B was frequently used as an effective elicitor to accelerate the biosynthesis of active ingredients (Morales et al., 2010). For examples, catharanthine of *Catharanthus roseus*, glycyrrhizin of *Glycyrrhiza uralensis*, chlorogenic acid of *Chrysanthemum morifolium* and taxol of *Taxus mairei*, were over-accumulated under the UV-B treatments (Afreen, Zobayed & Kozai, 2005; Ramani & Chelliah, 2007; Zu et al., 2010). Thus, UV-B might be also a good elicitor to accelerate the accumulation of active ingredients in *D. officinale*. However, the metabolic responses of *D. officinale* stems to UV-B radiation are largely unknown. Several comparative metabolomic analyses of *Dendrobium* have been performed in the past years. Metabolic profiles of *D. officinale* and *D. huoshanense* revealed their differences during different growth years (Jin et al., 2016a). Metabolic profiling of *D. officinale* provide a full view of the metabolic variations associated with alkaloid biosynthesis in response to MeJA treatment (Jiao et al., 2018). To understand the roles of UV-B in the biosynthesis of active ingredients of *D. officinale*, metabolic analysis was performed to reveal the variations in metabolite accumulation under the UV-B treatment.

## Materials & Methods

### Plant materials and UV-B treatment

Three-year-old *D. officinale* plants were used in this study. All the seedlings were planted in a growth chamber of Zhejiang Academy of Agriculture Science. The condition was set at a temperature of 25 ± 1 °C with a light/dark cycle of 12/12 h and 60%–70% relative humidity. For the UV-B treatment, 10 plants, as one group, were exposed to UV-B radiation for 12 h. The UV-B radiation was artificially generated by a UV-B lamp and wavelength of UV-B lower than 280 nm was filtered out by 3-mm transmission cutoff filters (Schott, Mainz, Germany). The irradiance of UV-B at the sampling area was set at 1.6 W m^−2^. The irradiance of UV-B was measured by an ultraviolet intensity meter (Apogee Instruments). Another 10 seedlings were planted in normal condition as the controls. Ten biological repetitions were applied to each group. Fresh stem samples were collected from the two groups of *D. officinale* seedlings after the treatments

### Metabolite isolation for untargeted metabolomic analyses

Stem samples from above two groups of *D. officinale* seedlings (50 mg each, *N* = 10) were harvested and put into different tubes. Each sample was added with 800 µL of pre-colded methanol (50%) and ground by a 2010 Geno/Grinder (SPEX SamplePrep, Metuchen, NJ, USA) at 1,900 strokes/min for 2 min. Then, the mixture was added with 500 µL of pre-colded chloroform/methanol/water (v:v:v, 1:3:1), shaken at 4 °C for 10 min, and put to ultrasonication for another 5 min. The supernatant was collected by 13,000× g centrifugation, vacuum-dried, and resuspended in methanol solution (50%). Control samples were arranged by mixing an equal volume of each experimental sample from different groups.

### UPLC-MS/MS analysis for the metabolomes

For the UPLC-MS/MS analysis, all samples were chromatographic fractionated using an Applied Biosystems SCIEX UPLC system (Foster City, CA, USA) with a Waters reversed-phase ACQUITY BEH Amide column (2.1 × 100 mm, 1.7 µm particle size; Milford, MA, USA). The temperature of oven was kept at 35  °CC and the rate of flow was set 0.4 mL/min. Mobile phase consisted of solution A and B. The solution A is an aqueous solution with 25 mM CH_3_COONH_4_ and 25 mM NH_4_H_2_O, and the solution B is a mixture of IPA:CAN (v:v, 9:1) adding with 0.1% formic acid. The gradient elution was set according to the previous study (Yu et al., 2018a). The fractions eluted from the column was subjected to a high-resolution MS/MS SCIEX Triple-TOF-5600 plus system and analyzed in both positive and negative ion modes. The detail parameters as set according to the previous study (Yu et al., 2018a).

### Bioinformatic analysis of the untargeted metabolomic dataset

Three softwares, including XCMSPlus software (https://sciex.com/products/software/xcms-plus-software), CAMMERA, and metaX toolbox (http://www.bioconductor.org/packages/2.4/bioc/html/CAMERA.html) were used to process the LC-MS/MS raw data (Smith et al., 2006). For MS data pretreatment, a series of operations, including peak and second peak grouping, peak picking, retention time (RT) correction, and annotation of isotopes and adducts, were carried out. Each ion was recognized by combining RT and *m/z* values. Intensity of each peak were calculated and a 3D matrix containing arbitrarily assigned peak indices (retention time-*m/z* pairs), sample names and ion intensity information was constructed.

The peak features that were detected in less than 50% of quality control (QC) samples or less than 80% of experimental samples were disregarded, the remaining peaks were processed using the K-nearest neighbor algorithm to improve their quality. Principal component analysis was carried out for outlier detection and batch effects evaluation. In addition, the relative standard deviations of the peak features were recorded across all the QC samples, and those more than 30% were deleted.

### Annotation of identified metabolites

The online Kyoto Encyclopedia of Genes and Genomes (KEGG, https://www.kegg.jp/) database was applied to annotate the metabolites by matching the *m/z* value of each sample with the metabolites from online database. If a mass difference in metabolite between the detected and the database value was <10 ppm, the metabolite would be annotated according the database and the its molecular formula would further be checked by the isotopic distribution measurements.

### Identification of differentially accumulated metabolites (DAMs) between groups

Wilcoxon tests were used to detect the differences in metabolite levels between two sample group. The *P* values were adjusted for multiple tests using an FDR with Benjamini–Hochberg method. Supervised partial least squares-discriminant analysis was applied through metaX software to discriminate the variables between two groups. A VIP cut-off value of 1.0 was used to screen key features. The differentially accumulated metabolites (DAMs) were selected with —fold change—>2 and with statistical significance (*P* value <0.05).

### Quantitative analysis of total alkaloids, polysaccharides and flavonoids

Several new seedlings were used in quantitative analysis. For quantitative analysis, three replicates were used. Stems from the seedlings under the two groups were harvested. The polysaccharide contents in different samples were determinated using the phenol-sulfuric acid method (Masuko et al., 2005). The total alkaloid contents were determinated using the UPLC method (Xu et al., 2010). In briefly, all stem samples were washed three times with 75% ethanol for 5 min, and heated for 2 h. The extracts were concentrated and dissolved with 5% hydrochloric acid. Then, aqueous acid solution was extracted three times by petroleum. The total alkaline solution was extracted three times with chloroform for the UPLC analysis.

Total flavonoids were determined by an aluminum nitrate colorimetric assay. In briefly, stem tissues were homogenized in 75% ethanol at room temperature for 30 min. The supernatant was extracted by 12,000× g centrifugation. A mixture solution of Al(NO_3_)_3_ and NaNO_2_ were added to the supernatant, and then coloration reaction was induced by addition of NaOH solution. Absorbance was determined at wavelength of 510 nm. Rutin was used as a standard.

Quantitative analysis of total alkaloids, polysaccharides and flavonoids was performed for five biological repeats and figures show the average values of five repeats.

### Real-time PCR validation

Transcriptomes of *D. officinale* were download from the NCBI database (SRR2014227). According to the transcriptomic annotation, four glycolysis-related genes, four flavonoid-related genes, and four alkaloid-related genes were selected. Sequence information of all these genes were Table S1. In the experiment of UV-B induced expression confirmation, a *D. officinale ACTIN* gene was used as an internal standard to analyze the related mRNA level basing on the comparative cycle threshold (2^−ΔΔ*Ct*^) values. Firstly, total RNAs were isolated from the samples used for the UV-B treatments. Total RNA of each sample was reverse transcribed to cDNA using a Thermo Scientific First Strand cDNA Synthesis Kit. Then, a SYBR Premix Ex Taq Kit and an ABI PRISM 7700 DNA Sequence Detection System were used for qRT-PCR analysis. Three independent samples of each treatment were used for the qRT-PCR analysis. The saw data and primer sequences are showed in Table S1. Differences in values between two groups were calculated using one-way ANOVA with Student’s *t*-test at *P* < 0.05.

## Results

### Untargeted metabolomes of *D. officinale* under the control and UV-B treatment

**Figure 1:**
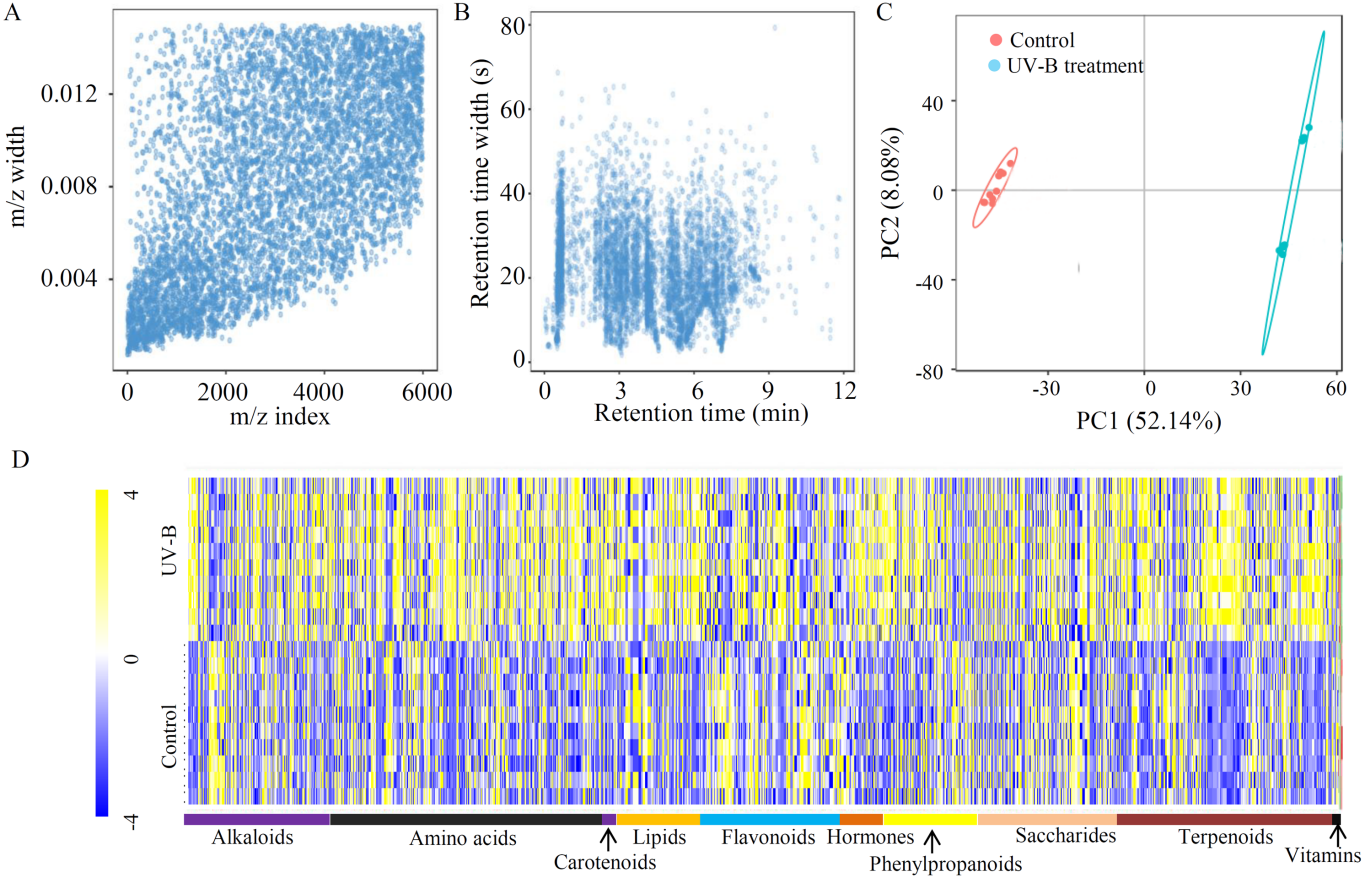
Untargeted metabolite profiling identifies the metabolites of *D. officinale* under the control and UV-B treatments. (A) The *m/z* width of the MS data. (B) The retention time width of the MS data. (C) The PCA data of the samples from two different groups. (D) A heatmap of the metabolites classed into various major primary metabolic categories (*N* = 10). The heatmap scale ranges from −4 to +4 on a log_2_ scale.

To explore the effects of UV-B treatment on the accumulation of bioactive ingredients in *D. officinale*, an untargeted approach was applied, identifying 3,655 metabolites from 5,994 ion features (Table S2). To check the quality of the MS data, *m/z* width and RT width were analyzed, suggesting that instrument preparation reached the standards (Figs. 1A and 1B). A PCA showed that the percentages of value in the metabolite analysis of PC1 and PC2 were 52.14% and 8.08%, respectively (Fig. 1C). Based on the annotations, many metabolites were grouped into at least one KEGG category. The top five largest KEGG categories were ‘Global and overview maps’ (757 metabolites), ‘Biosynthesis of other secondary metabolites’ (315 metabolites), ‘Amino acid metabolism’ (211 metabolites), ‘Carbohydrate metabolism’ (177 metabolites), and ‘Metabolism of terpenoids and polyketides’ (158 metabolites) (Fig. S1). Furthermore, 1403 metabolites were grouped into 10 primary metabolic categories, including alkaloids (176 metabolites), amino acids (330 metabolites), carotenoids (17 metabolites), lipids (102 metabolites), flavonoids (169 metabolites), hormones (52 metabolites), phenylpropanoids (112 metabolites), saccharides (168 metabolites), terpenoids (267 metabolites), and vitamins (10 metabolites) (Fig. 1D and Table S3).

### Identification of DAMs between the control and UV-B treatment groups

**Figure 2:**
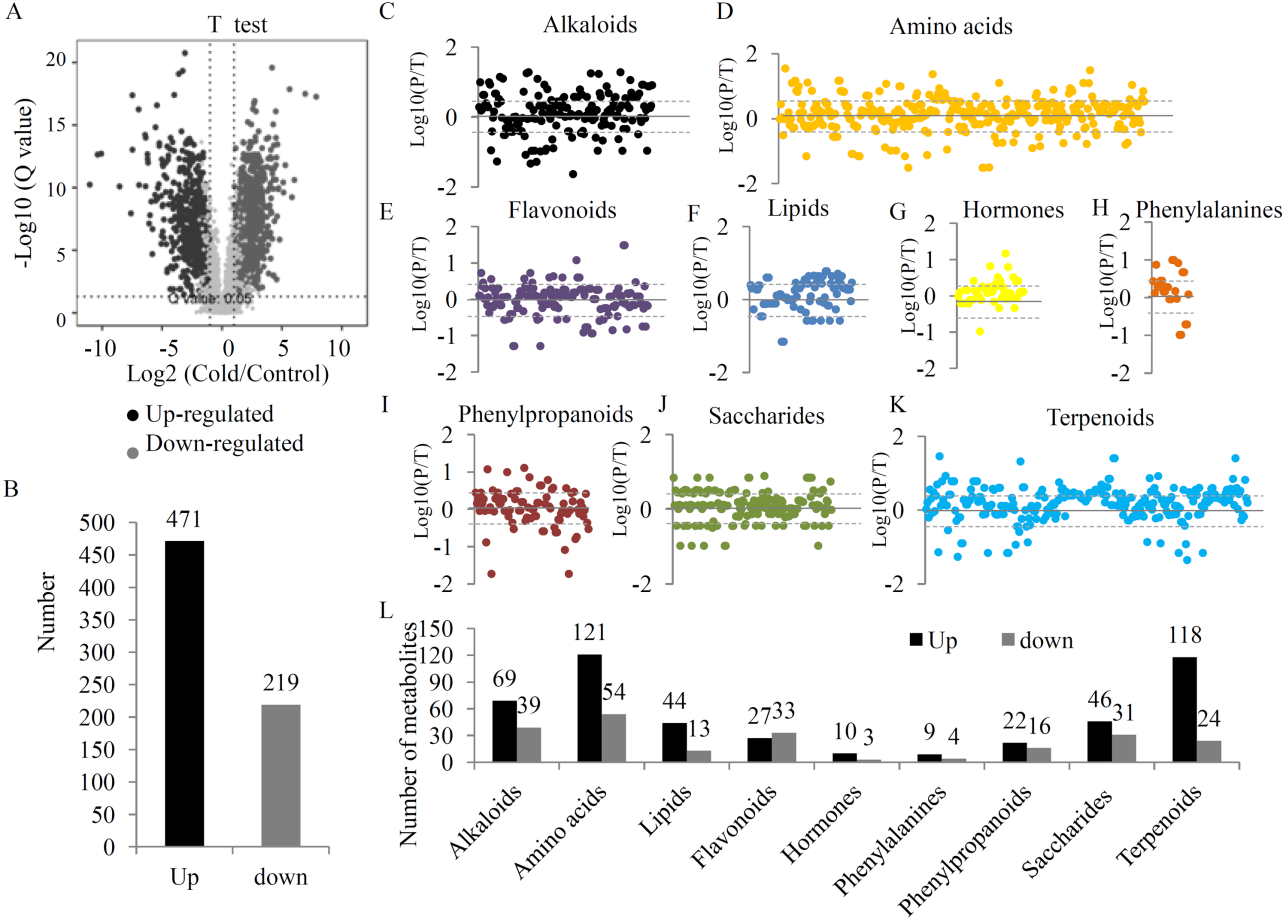
The variations in the metabolites between two treatment groups. (A) Significance analysis of the DAMs between the two treatment groups by Volcanoplot. (B) The numbers of up- and down-regulated metabolites under the UV-B treatment. DAMs were grouped into different primary metabolic categories, including (C) alkaloids, (D) amino acids, (E) flavonoids, (F) lipids, (G) hormones, (H) phenylalanines, (I) phenylpropanoids, (J) saccharides, (K) terpenoids. (L) The detection limit of DAMs was indicated by dotted line. The value scale ranges from -2 to +2 on a log_2_ scale. (L) The numbers of up- and down-regulated metabolites belonging to each primary metabolic category.

To provide a comprehensive overview of metabolic variations under the UV-B treatment, two quality control parameters, coefficient of variation (CV) and normalized intensity, were checked. Our data showed a obvious separation between the two groups (Figs. S2 and S3). After filtering, 4827 high quality metabolites were selected to analyze the DAMs between the two groups (Table S4). Statistical analysis showed 1423 significant DAMs, including 640 up- and 783 down-regulated metabolites (Figs. 2A, 2B and Table S5). A total of 690 DAMs were grouped into different primary metabolic categories, including 176 alkaloids, 303 amino acids, 102 lipids, 169 flavonoids, 52 hormones, 25 phenylalanines, 87 phenylpropanoids, 168 saccharides, and 267 terpenoids (Fig. 2C). For several primary metabolic categories, the number of up-regulated metabolites was larger than that of down-regulated metabolites. For example, 121 up- and 54 down-regulated amino acids, 44 up- and 13 down-regulated lipids, 118 up- and 24 down-regulated terpenoids, and 46 up- and 31 down-regulated saccharides were identified under the UV-B radiation (Fig. 2D).

### Putative metabolites associated with alkaloid biosynthesis of *D. officinale*

**Figure 3:**
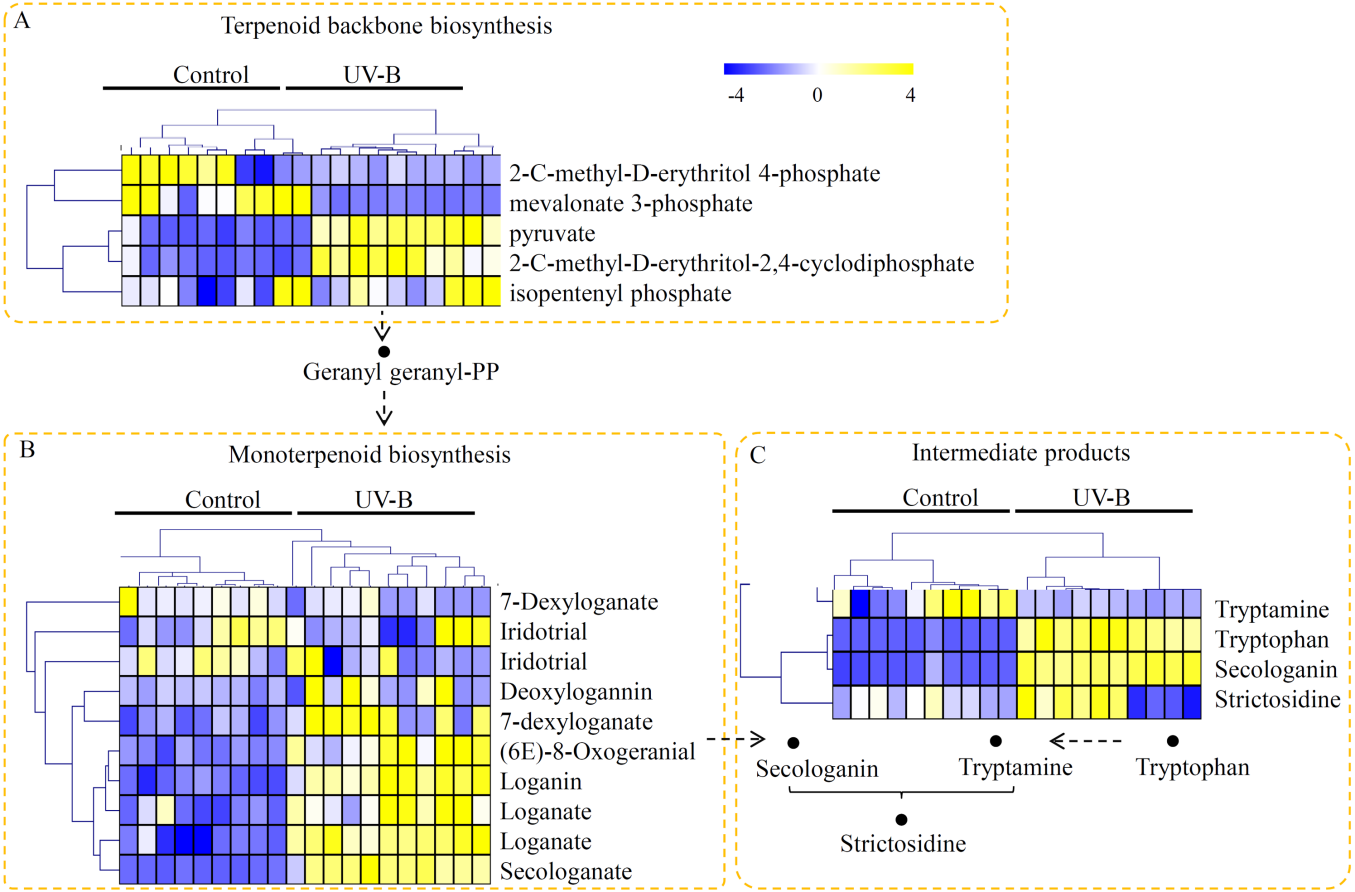
Untargeted metabolite profiling identifies the metabolites of *D. officinale* under the control and UV-B treatments. (A) The *m/z* width of the MS data. (B) The retention time width of the MS data. (C) The PCA data of the samples from two different groups. (D) A heatmap of the metabolites classed into various major primary metabolic categories (*N* = 10). The heatmap scale ranges from −4 to +4 on a log_2_ scale.

The primary style of alkaloids in *D. officinale* was TIA, and therefore the metabolites assigned into the TIA biosynthesis pathway were screened. In our study, the metabolites classified into the putative upstream elements of alkaloid biosynthetic pathway, including ‘’terpenoid backbone biosynthesis’, ‘monoterpenoid biosynthesis’, and ‘intermediate products’, were identified. In detail, five metabolites, including 2-C-methyl-D-erythritol 4-phosphate, mevalonate 3-phosphate, pyruvate, 2-C-methyl-D-erythritol-2,4-cyclodiphosphate, and isopentenyl phosphate, were targeted to the terpenoid backbone biosynthesis pathway. Among the metabolites in terpenoid backbone biosynthesis pathway, three metabolites were predominantly accumulated under the UV-B treatment (Fig. 3A). For the monoterpenoid biosynthesis pathway, eight metabolites, including 7-dexyloganate, iridotrial, deoxylogannin, 7-dexyloganate, (6E)-8-oxogeranial, loganin, loganate, and secologanate, were identified, five of which were significantly up-regulated by the UV-B treatment (Fig. 3B). Moreover, four key intermediate products, such as tryptamine, tryptophan, secologanin, and strictosidine, were identified. Interestingly, three of the four key intermediate products were highly accumulated under the UV-B treatment (Fig. 3C).

### Putative metabolites associated with polysaccharide biosynthesis of *D. officinale*

**Figure 4:**
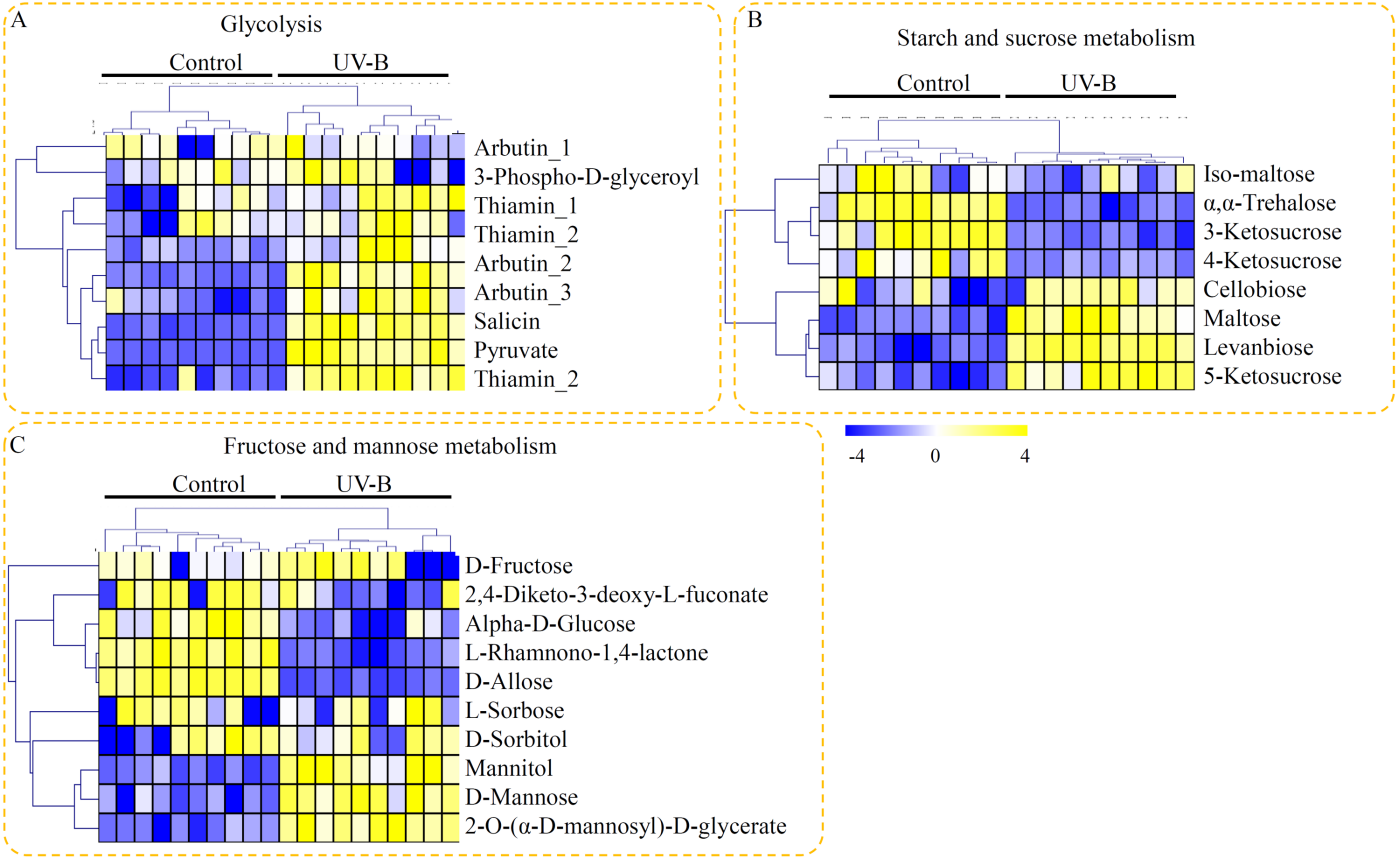
Putative metabolites associated with polysaccharide biosynthesis of *D. officinale*. (A) Metabolites associated with the glycolysis pathway. (B) Metabolites associated with the starch and sucrose metabolism pathway. (C) Metabolites associated with the fructose and mannose metabolism pathway. The heatmaps showed the relative amounts of metabolites from the two treatment groups. The heatmap scale ranges from −4 to +4 on a log_2_ scale.

Based on their KEGG annotation, three metabolic pathways, providing fundamental building blocks for polysaccharide biosynthesis, were identified in *D. officinale*. For the glycolysis, nine metabolites, including seven UV-B up-regulated metabolites, were identified (Fig. 4A). For the starch and sucrose metabolism pathway, eight metabolites, including iso-maltose, α, α-trehalose, 3-ketosucrose, 4-ketosucrose, cellobiose, maltose, levanbiose, and 5-ketosucrose, were identified. Half of the metabolites involved in the starch and sucrose metabolism pathway were up-regulated by the UV-B treatment (Fig. 4B). For the fructose and mannose metabolism pathway, ten metabolites, including D-fructose, 2,4-diketo-3-deoxy-L-fuconate, D-mannose, L-rhamnono-1,4-lactone, D-allose, D-sorbitol, mannitol, α-D-glucose, and 2-O-(α-D-mannosyl)-D-glycerate, were identified. Among these fructose and mannose metabolism-related metabolites, only three metabolites (mannitol, D-mannose, and 2-O-(α-D-mannosyl)-D-glycerate) were significantly up-regulated by the UV-B treatment (Fig. 4C).

### Putative metabolites associated with flavonoid biosynthesis of *D. officinale*

**Figure 5:**
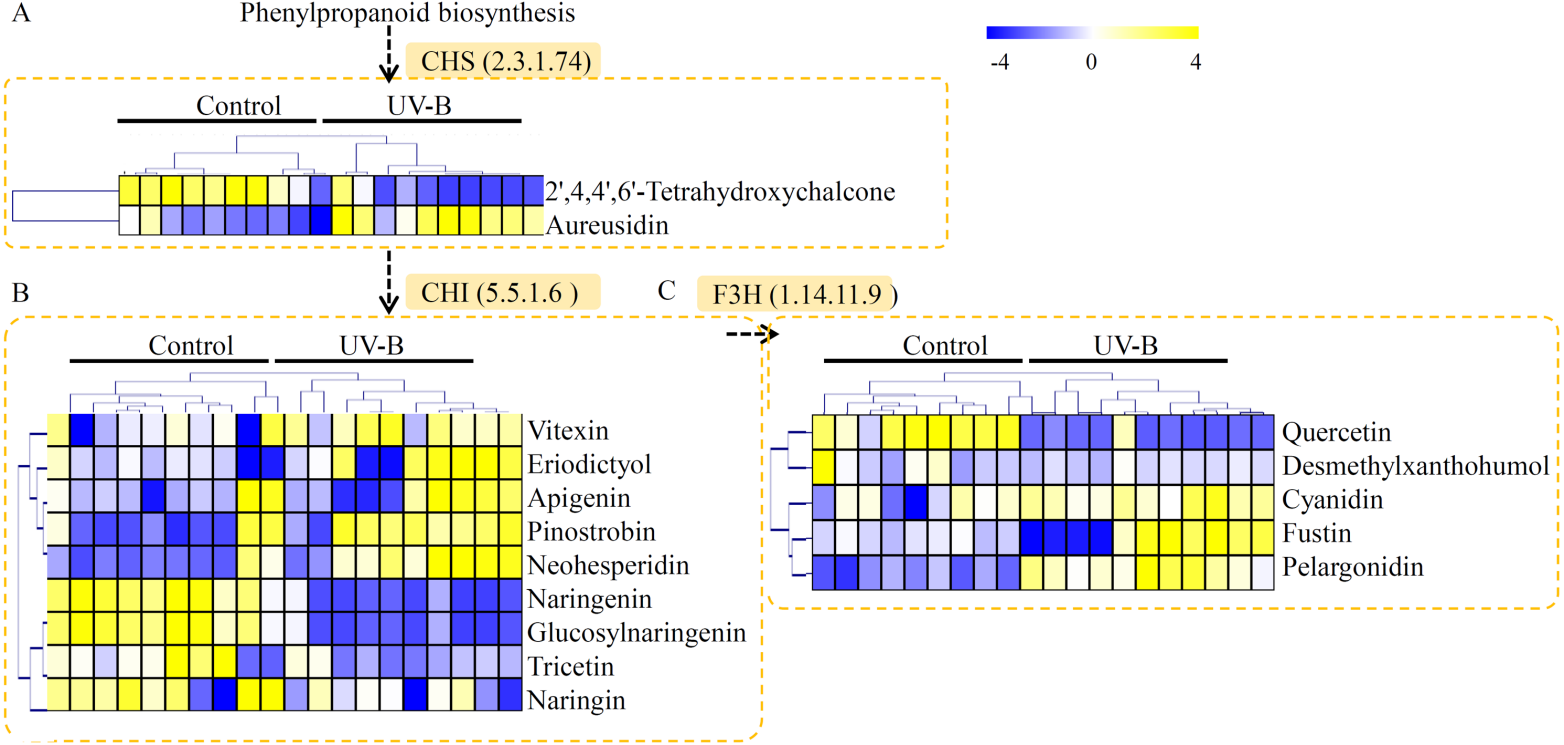
Putative metabolites associated with flavonoid biosynthesis of *D. officinale*. (A) The downstream products of CHS. (B) The downstream products of CHI. (C) The downstream products of F3H. The heatmaps showed the relative amounts of metabolites from the two treatment groups. The heatmap scale ranges from −4 to +4 on a log_2_ scale.

In our study, many metabolites related to flavonoid metabolism were identified in *D. officinale*, including two products of CHALCONE SYNTHASE (CHS), nine products of CHALCONE ISOMERASE (CHI), and five products of FLAVANONE 3-HYDROXYLASE (F3H). As the downstream products of CHS, 2′,4,4′,6′-tetrahydroxychalcone was down-regulated and aureusidin was induced by the UV-B treatment (Fig. 5A). As the downstream products of CHI, five metabolites, including vitexin, eriodictyol, apigenin, pinostrobin, and neohesperidin, were significantly accumulated in the seedlings under UV-B treatment (Fig. 5B). As the downstream products of F3H, three metabolites, including cyanidin, fustin, and pelargonidin, were greatly up-regulated by the UV-B treatment (Fig. 5C).

### Determination of total polysaccharides, total alkaloids and total flavonoids

**Figure 6:**
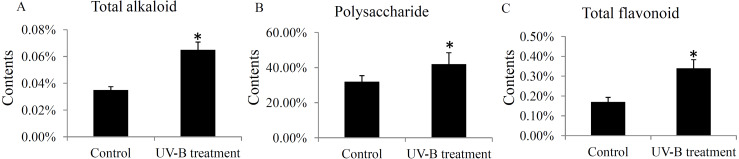
Variation of the major active ingredients of *D. officinale* under different treatment conditions. The contents of total alkaloids (A), polysaccharides (B) and flavonoids (C) were quantified by HPLC analysis. A *P* value < 0.05 was considered to be statistically significant and indicated by “*”.

The contents of total alkaloids, total polysaccharides, and total flavonoids were determinated in the control and UV-B treatment groups. The contents of total alkaloids were induced from 0.035% to 0.065% by the UV-B treatment (Fig. 6A). The contents of total polysaccharides were up-regulated from 32% to 42% by the UV-B treatment (Fig. 6B). Moreover, the contents of total flavonoids were increased from 0.17% to 0.34% by the UV-B treatment (Fig. 6C). The saw data was listed in Table S6.

### Validation of the expression levels of several genes associated with bioactive ingredients

**Figure 7:**
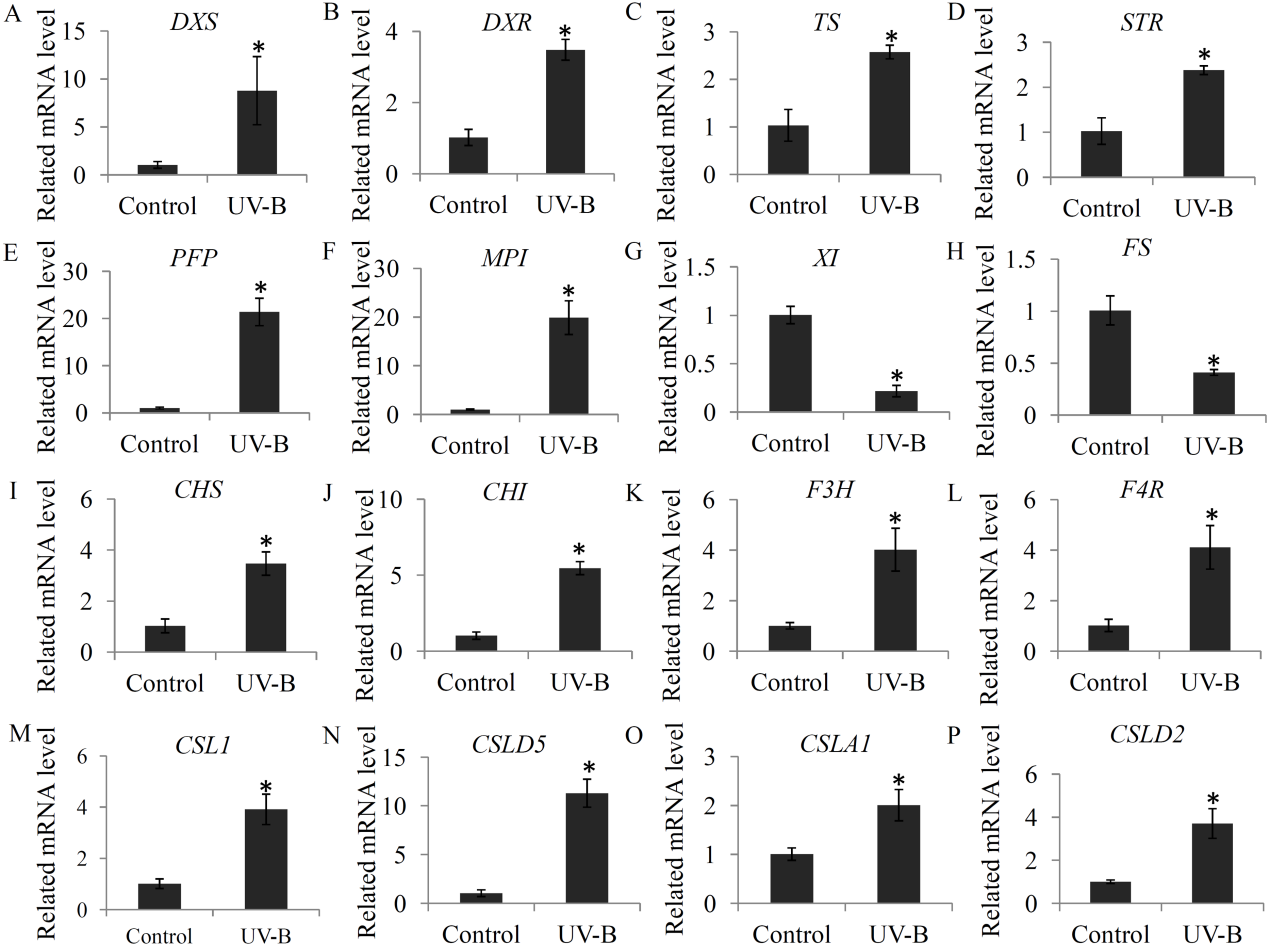
Validation of the expression levels of several genes associated with bioactive ingredients. (A–D) The expression levels of four alkaloid biosynthesis-related genes; (E–H) The expression levels of four polysaccharide biosynthesis-related genes; (I–L) The expression levels of four flavonoid biosynthesis -related genes. Gene abbreviations are: 1-Deoxy-D-xylulose-5-phosphate synthase (DXS), 1-Deoxy-D-xylulose-5-phosphate reductoisomerase (DXR), Tryptophan synthase (TS), Strictosidine synthase (STR), Pyrophosphate-fructose 6-phosphate 1-phosphotransferase (PFP), Mannose-6-phosphate isomerase (MPI), Xylose isomerase (XI), GDP-L-fucose synthase (FS), Chalcone synthase (CHS), Chalcone isomerase (CHI), Flavanone 3-hydroxylase (F3H), and Flavanone 4-reductase (F4R). Each bar shows the mean ±SD (*n* = 3) of triplicate assays. The significantly changes (*P* < 0.05) in the contents between the treatments and control were indicated by “*”.

To check the changes in the expression levels of several genes associated with bioactive ingredient biosynthesis, a qRT-PCR assay was performed. For the glycosylsis, four genes, including pyrophosphate-fructose 6-phosphate 1-phosphotransferase (*PFP*), mannose-6-phosphate isomerase (*MPI*), xylose isomerase (*XI*), GDP-L-fucose synthase (*FS*), were selected; for alkaloid biosynthesis, four key genes, including 1-deoxy-D-xylulose-5-phosphate synthase (*DXS*), 1-deoxy-D-xylulose-5-phosphate reductoisomerase (*DXR*), tryptophan synthase (*TS*), and strictosidine synthase (*STR*), were selected; for flavonoid biosynthesis, four key genes, including *CHS*, *CHI*, *F3H*, and flavanone 4-reductase (*F4R*), were selected; and for polysaccharide biosynthesis, four genes, including cellulose synthase-like 1 (*CSL1*), cellulose synthase-like D5 (*CSLD5*), cellulose synthase-like A1 (*CSLA1*), and cellulose synthase-like D3 (*CSLD3*), were selected. Our data showed that the selected flavonoid biosynthesis- and alkaloid biosynthesis-related genes were siginificantly induced by the UV-B treatment. For the glycosylsis-related genes, the *PFP* and *MPI* genes were up-regulated and the *XI* and *FS* genes were reduced by the UV-B treatment. For the polysaccharide biosynthesis-related genes, *CSL1*, *CSLD5*, *CSLA1* and *CSLD2* were significantly up-regulated by the UV-B treatment (Fig. 7).

## Discussion

The genus *Dendrobium*, consisting of many ornamental plants and medicinal herbs, is the largest orchid genera with over 1,000 species (Jin, Chen & Luo, 2009). *D. officinale* is considered to be a traditional Chinese medicine with increasing commercial value. Recently, the biological activities of the extracted polysaccharides, flavonoids and alkaloids have been well studied. However, the industrial application and quality evaluation of *D. officinale* remains a great challenge due to the unstable contents and unclear biosynthetic pathways of its active ingredients (Guo et al., 2013; Ng et al., 2012).

The effects of UV-B radiation on the primary and secondary active ingredients in various plant species have been widely reported. For example, a low dose of UV-B radiation for short-term treatment stimulates the biosynthesis of artemisinin in the *Artemisia annua* L. seedlings (Pan et al., 2014). In grapevine berries, UV-B radiation alters flavonol and anthocyanin profiles through regulating a series of flavonoid hydroxylase genes (Martinez-Luscher et al., 2014). In *Chrysanthemum morifolium* leaves, the levels of flavonoids, caffeoylquinic acids and fatty acids were up-regulated under the UV-B treatment (Yang et al., 2018). Our data showed that the total contents of alkaloids, polysaccharides, and flavonoids were significantly increased under the UV-B treatment (Fig. 6), confirming the effects of UV-B radiation on the ingredients accumulation in *D. officinale* stems.

Untargeted metabolic analysis was a newly developed technology for systematically comparison of the primary and secondary metabolites in plants under different conditions (Yu et al., 2018a; Zhan et al., 2018). Recently, several metabolomes of *Dendrobium* genus have be reported. By Cai’s group, a metabolic analysis has identified a series of biomarkers that discriminate between *D. officinale* and *D. huoshanense* (Jin et al., 2016a). By Wu’s group, a large scale metabolomic analysis revealed a global reprogramming of metabolic regulation networks of *D. officinale* during cold acclimation (Wu et al., 2016). By Jin’s group, metabolic profiling of *D. officinale* in response to tryptophan, secologanin and MeJA treatments provide important clues for exploring the molecular mechanism of TIA biosynthesis regulation (Jiao et al., 2018). In our study, 3655 metabolites with annotation were identified (Table S2), which was significantly larger than the numbers of identified metabolites in Jin’s study (78 metabolites), in Wu’s study (68 metabolites), and in Cai’s study (139 metabolites) (Jiao et al., 2018; Jin et al., 2016a; Wu et al., 2016). Embracing comprehensive metabolite profiling gave us an opportunity to explore the mechanism underlying the UV-B-induced active ingredients accumulation in *D. officinale* stems.

In plants, expression of terpenoid backbone biosynthesis-related genes, such as 2-C-methyl-D-erythritol-2,4-cyclodiphosphate synthase encoding gene (*MDS*), and isopentenyl diphosphate isomerase encoding gene (*IPPI*), was modulated by the UV-B treatment (Dolzhenko et al., 2010). In *D. officinal* e, expression of two initiating genes in the terpenoid backbone biosynthesis pathway, *DXS* and *DXR*, were significantly up-regulated by the UV-B treatment (Fig. 7). Moreover, 2-C-methyl-D-erythritol-2,4-cyclodiphosphate and isopentenyl phosphate were significantly induced by the UV-B treatment, suggesting that the precursors of alkaloid biosynthesis are abundant in the stems under the UV-B treatment. Beside, strictosidine, which is synthesized by tryptamine and secologanin, is the key intermediate product of the biosynthesis of alkaloid backbone in various plants (Guo et al., 2013; McKnight et al., 1990). Many previous studies showed that the secologanin and tryptamine precursors mostly derived from the monoterpenoid biosynthesis pathway (De Luca et al., 2014). In plants, TS catalyzes the final two steps of tryptophan biosynthesis (Jin et al., 2016b). Under UV-B treatment, increasing in expression of TS encoding gene might ensure the adequately supplying of tryptamine precursor. STR functions as an essential factor invloved in the biosynthesis of terpenoid indole alkaloids and is produced in many active meristematic organs of different plants (Sohani et al., 2009). In *D. officinale*, the encoding gene of STR was siginificantly up-regulated by the UV-B treatment (Fig. 7), suggesting an enhanced terpenoid indole alkaloid pathway activity. Interestingly, most of the intermediate metabolites in monoterpenoid biosynthesis pathway were highly accumulated under the UV-B treatment (Fig. 3B). Our data suggested that UV-B radiation played a positive role in the UV-B-induced accumulation of alkaloids by up-regulating their precursors and intermediate metabolites.

Polysaccharides, highly accumulated in the stems of *D. officinale*, are one of the major active constituents for drug uses (He et al., 2015). In *D. officinale*, glycolysis is the upstream metabolic pathway of polysaccharide biosynthesis (Feng et al., 2017). Oxidization of glucose to pyruvate is a central metabolic pathway (Zhao & Assmann, 2011). In our study, most of the metabolites involved in glycolysis, particularly pyruvate, were significantly up-regulated by the UV-B treatment, suggesting a positive effect of UV-B radiation on the glycolysis pathway (Fig. 4). Chemical analysis showed that *D. officinale* polysaccharides were composed of mannose, glucose and arabinose, which were the fundamental blocks for the biosynthesis of polysaccharides in *D. officinale* (He et al., 2016; Luo et al., 2016). In our study, D-mannose was significantly induced by the UV-B treatment (Fig. 4C). Furthermore, qRT-PCR data showed that *PFP* and *MPI* genes, which encode two key enzymes involving in the glycolysis pathway, were significantly up-regulated under the UV-B treatment (Fig. 7). In plants, PFP regulates carbon metabolism and MPI affects sucrose metabolism (Duan et al., 2016; Zhang et al., 2015). Our results suggested an essential role of UV-B in the accumulation of building materials of polysaccharide biosynthesis.

Addition to polysaccharides and alkaloids, several individual flavonoids, such as naringenin, flavonoid C-glycoside and flavonoid O-glycoside, were also important biomarkers for dendrobium species discriminant (Chen et al., 2012; Shen et al., 2012). To date, increasing evidences uncovered the close relationship between UV-B radiation and flavonoid biosynthesis (Fraser et al., 2017). For example, expression of several flavonoid biosynthesis pathway-related genes, including *CHS*, *CHI*, *F3H*, was induced by the UV-B radiation (Christie & Jenkins, 1996). Basing on the previous published transcriptomes, four key flavonoid biosynthesis-related genes were isolated in *D. officinale*. The expression of *CHS*, *CHI*, *F3H*, and *F4R* were significantly induced by the UV-B treatment, indicating an involvement of UV-B radiation in flavonoid biosynthesis. In *D. officinale*, many intermediate products of CHS, CHI and F3H enzymes were up-regulated by the UV-B treatment, (Fig. 5). Increasing in the expression of flavonoid biosynthesis-related genes might be an exciting cause of the UV-B induced flavonoid accumulation. In plants, flavonoids were considered to be a class of active constituents with antioxidant activities and play important roles in the responses to environmental stresses (Hao et al., 2017; Yang et al., 2018). Increasing in the accumulation of flavonoids might help *D. officinale* seedlings to avoid UV-B radiation caused damages. Moreover, many other intermediate metabolites in flavonoid biosynthesis pathway were down-regulated by the UV-B treatment, indicating a complex response of flavonoid metabolism to UV-B radiation in *D. officinale*.

## Conclusions

A untargeted method was used to investigate the metabolic variations in the *D. officinale* stems under UV-B radiation. In total, 3,655 annotated metabolites, including 640 up- and 783 down-regulated metabolites, were identified. Most of the metabolites involved in alkaloid biosynthesis (secologanate and strictosidine), polysaccharide biosynthesis (D-mannose and pyruvate) and flavonoid metabolism (apigenin and cyanidin) were significantly up-regulated by the UV-B treatment. Furthermore, the UV-B induced active ingredient accumulations were confirmed by a target UPLC analysis. Our study will help understand the regulation mechanism underlying the UV-B-induced active ingredient accumulation in the stems of *D. officinale*.

##  Supplemental Information

10.7717/peerj.9107/supp-1 Figure S1 KEGG analysis of all the identified metabolites in D. officinale under the control and UV-B treatment

10.7717/peerj.9107/supp-2 Figure S2 Analysis of the coefficient of variation values

10.7717/peerj.9107/supp-3 Figure S3 Analysis of the normalized intensity values

10.7717/peerj.9107/supp-4 Table S1 The raw data of the qRT-PCR analysis

10.7717/peerj.9107/supp-5 Table S2 The detailed information of the untargeted metabolomes of D. officinale under the control and UV-B treatment

10.7717/peerj.9107/supp-6 Table S3 The detailed information of the 1403 metabolites grouped into 10 primary metabolic categories

10.7717/peerj.9107/supp-7 Table S4 The detailed information of all metabolites between the control and UV-B treatment

10.7717/peerj.9107/supp-8 Table S5 The detailed information of DAMs between the control and UV-B treatment

10.7717/peerj.9107/supp-9 Table S6 The raw data of the total polysaccharides, total alkaloids and total flavonoids

## Additional Information and Declarations

### Data Availability

The following information was supplied regarding data availability: The raw data are available in Table S1 and S2.
