# Supplementary material for: Comparative metabolomic analyses of Dendrobium officinale Kimura et Migo responding to UV-B radiation reveal variations in the metabolisms associated with its bioactive ingredients
Source: PeerJ. 2020 Jun 29;8:e9107. doi: 10.7717/peerj.9107 (PMC7331624; doi:10.7717/peerj.9107)
Supplement: Figure S1 [file peerj-08-9107-s001.pdf]

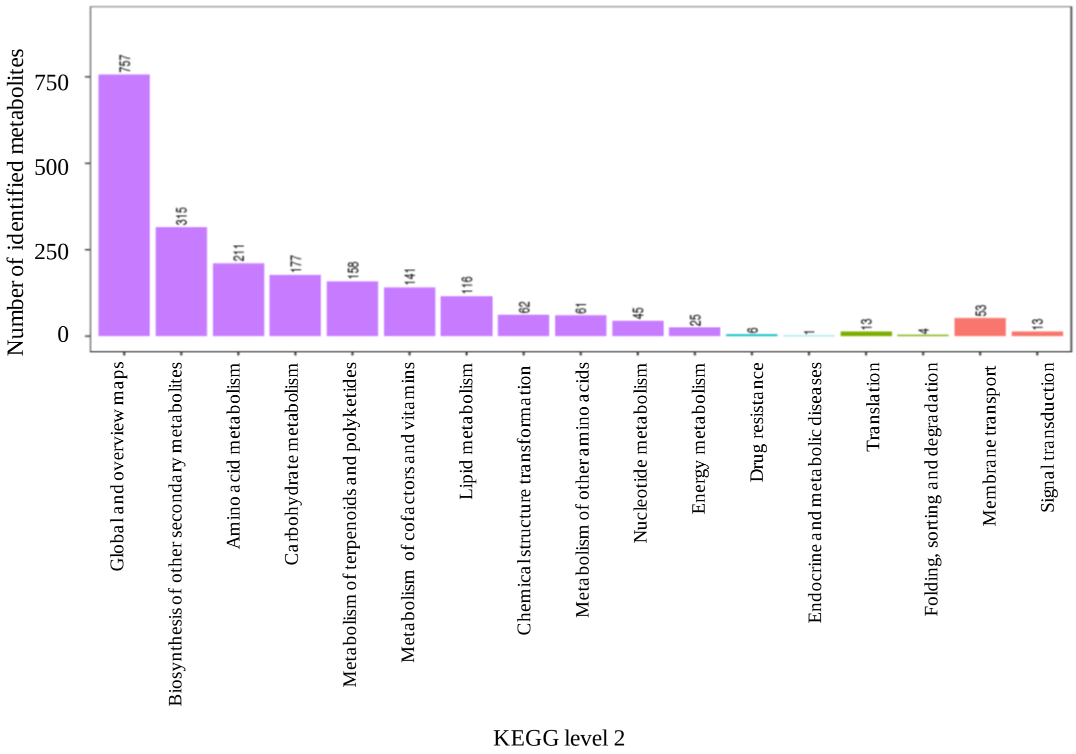

Figure S1 KEGG analysis of all the identified metabolites in *D. officinale* under the control and UV-B treatment.
