# Supplementary figures and images for: Comparative metabolomic analyses of Dendrobium officinale Kimura et Migo responding to UV-B radiation reveal variations in the metabolisms associated with its bioactive ingredients
Source: PeerJ. 2020 Jun 29;8:e9107. doi: 10.7717/peerj.9107 (PMC7331624; doi:10.7717/peerj.9107)

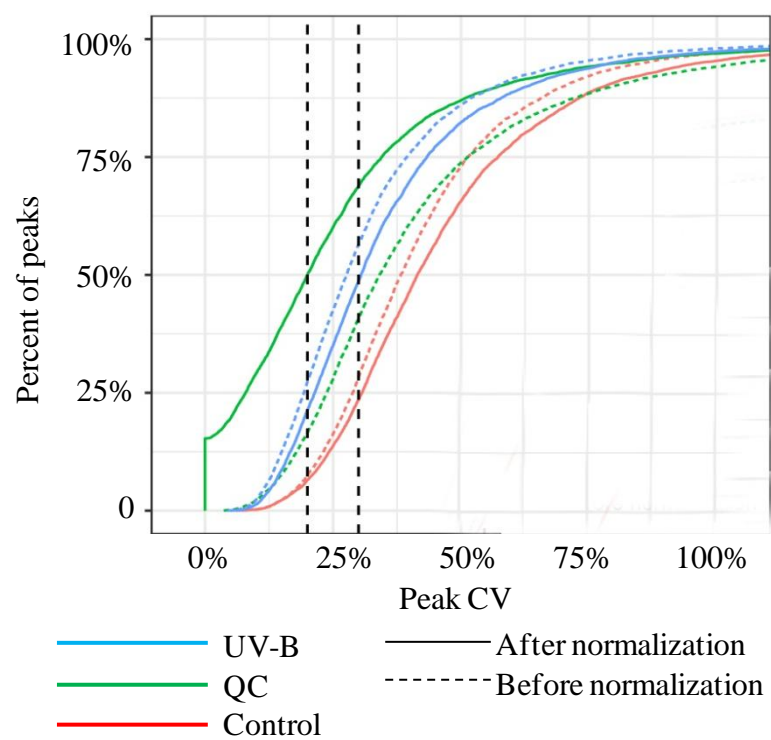

Figure S2 Analysis of the coefficient of variation values.

Supplement: Figure S2 [file peerj-08-9107-s002.pdf]

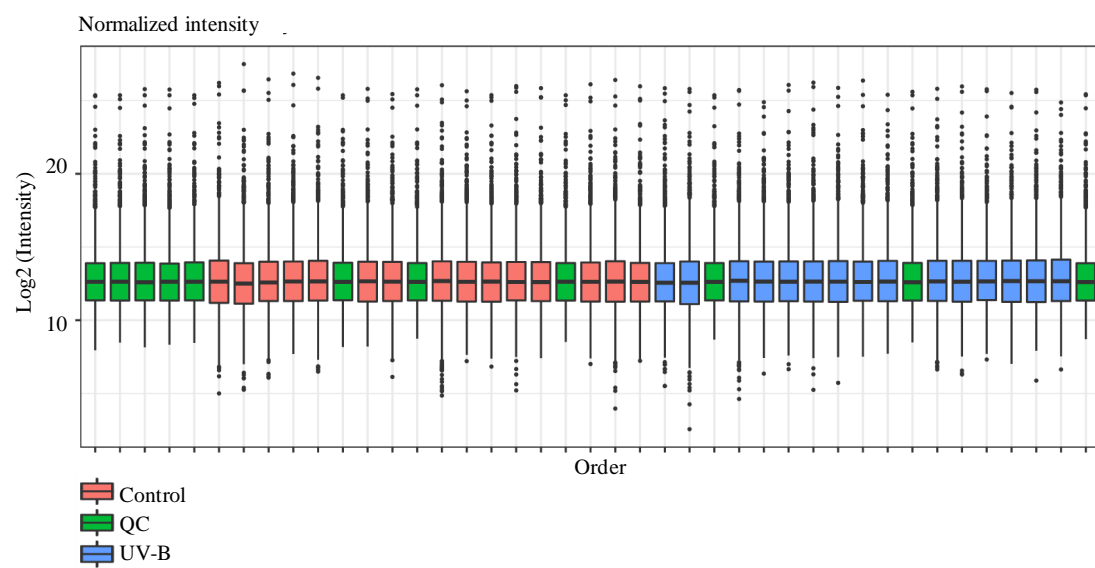

Figure S3 Analysis of the normalized intensity values.

Supplement: Figure S3 [file peerj-08-9107-s003.pdf]
